# A Novel Automatic Detection System for ECG Arrhythmias Using Maximum Margin Clustering with Immune Evolutionary Algorithm

**DOI:** 10.1155/2013/453402

**Published:** 2013-04-18

**Authors:** Bohui Zhu, Yongsheng Ding, Kuangrong Hao

**Affiliations:** ^1^College of Information Sciences and Technology, Donghua University, Shanghai 201620, China; ^2^Engineering Research Center of Digitized Textile & Fashion Technology, Ministry of Education, Donghua University, Shanghai 201620, China

## Abstract

This paper presents a novel maximum margin clustering method with immune evolution (IEMMC) for automatic diagnosis of electrocardiogram (ECG) arrhythmias. This diagnostic system consists of signal processing, feature extraction, and the IEMMC algorithm for clustering of ECG arrhythmias. First, raw ECG signal is processed by an adaptive ECG filter based on wavelet transforms, and waveform of the ECG signal is detected; then, features are extracted from ECG signal to cluster different types of arrhythmias by the IEMMC algorithm. Three types of performance evaluation indicators are used to assess the effect of the IEMMC method for ECG arrhythmias, such as sensitivity, specificity, and accuracy. Compared with *K*-means and iterSVR algorithms, the IEMMC algorithm reflects better performance not only in clustering result but also in terms of global search ability and convergence ability, which proves its effectiveness for the detection of ECG arrhythmias.

## 1. Introduction

Electrocardiogram (ECG) is widely used in cardiology since it consists of effective, simple, noninvasive, low-cost procedures for the diagnosis of cardiovascular diseases (CVDs). Since the state of cardiac heart is generally reflected in the shape of ECG waveform and heart rate, ECG is considered to be a representative signal of cardiac physiology, useful in diagnosing cardiac disorders and detecting any arrhythmia [1, 2]. 

ECG arrhythmia can be defined as any of a group of conditions in which the electrical activity of the heart is irregular and can cause heartbeat to be slow or fast. It can take place in a healthy heart and be of minimal consequence, but they may also indicate a serious problem that leads to stroke or sudden cardiac death. As ECG signal being nonstationary signal, the arrhythmia may occur at random in the time-scale, which means, the arrhythmia symptoms may not show up all the time but would manifest at certain irregular intervals during the day. Therefore, for effective diagnostics, the variability of ECG signal may have to be observed over several hours. For this reason, together with the fact that the volume of the ECG data is enormous, the study is tedious and time consuming. Thus, automatic and computer-based detection and classification of arrhythmia is critical in clinical cardiology, especially for the treatment of patients in the intensive care unit [1].

In the recent years, several methods have been developed in the literatures for detection and classification of ECG arrhythmias. Artificial neural network (ANN) classification method is one of the main methods for ECG arrhythmia recognition. By integration of many data reduction and feature extraction techniques, such as principal component analysis (PCA), independent component analysis, fuzzy logic, and wavelet transform (WT), improved ANN techniques have been shown to be able to recognize and classify ECG arrhythmia accurately [3–7]. However, many ANN algorithms suffer from slow convergence to local and global minima and from random settings of initial values of weights [7]. Since support vector machine (SVM) classifiers do not trap in local minima points and need less training input, various methods of SVM have been adopted for ECG signals classification and proved to be effective [8–11].

Although many ECG arrhythmia classification methods show good performance in the laboratory, there are only few techniques gaining popularity in practical applications. One of the main reasons is that most methods are supervised methods which require multiple samples manually labeled with the correct type of ECG signals in context. From these samples, a supervised system can learn to predict the correct sense of the similar ECG signal in a new context. However, these data sets are labor intensive, time consuming, and expensive to produce; thus, few data could be labeled and may be only for several ambiguous types. Therefore, using this technique to detect all kinds of arrhythmias is not optimal in the diagnosis of cardiovascular arrhythmia. Moreover, same state of cardiac heart presents different ECG waveforms for different individual characteristics because of the differences in their body, such as heart volume and coronary artery. Even for the same individual, the waveforms would present different shapes when the sample is involved in different activity states, such as walking, running, and sleeping. In order to address this problem, some methods containing unsupervised techniques are developed to analyze the ECG arrhythmia [4–6, 12–16], which do not need any labeled training sample and can find out unknown ECG arrhythmia. In these methods, the key point is the design of an ideal clustering method, as the accuracy of cluster analysis significantly affects the overall performance.

In this paper, we propose a novel immune evolution maximum margin clustering method (IEMMC) for ECG arrhythmias detection. Specifically, we decompose the ECG arrhythmias diagnosis procedure into three steps, including signal processing, feature extraction, and clustering. First, we apply a wavelet transform based adaptive filter to remove the noise and detect ECG waveform. Then, features are extracted to represent ECG signal. Finally, we employ maximum margin clustering (MMC) method to recognize ECG arrhythmias. Considering huge amount of ECG data and expensive computation of traditional MMC algorithm [17], we propose the IEMMC algorithm as the improvement of the existing MMC and make it more suitable for the detection of ECG abnormalities. Our key contribution is to utilize immune evolutionary algorithm to perform optimization directly on the nonconvex optimization problem formulated by original MMC problem and find the optimal solution which has maximum margin. Our IEMMC method avoids the requirement of solving a nonconvex integer problem and semidefinite programming (SDP) relaxations in the traditional MMC algorithm, which is computationally expensive and time consuming. Due to the outstanding global search ability and robustness of immune evolutionary algorithm, performance of the IEMMC algorithm could maintain at a high level even with a poor quality of random initialization, and the astringency of the IEMMC method is also superior to the existing approaches.

The rest of this paper is organized as follows.  Section 2 describes our proposed ECG arrhythmias detection system, including signal preprocessing, feature extraction, and the IEMMC method for ECG arrhythmias. Then, the cluster performance is examined through simulation experiments in Section 3. Finally, the concluding remarks are given in Section 4.

## 2. A Novel Automatic Detection System for ECG Arrhythmias

**Figure 1:**
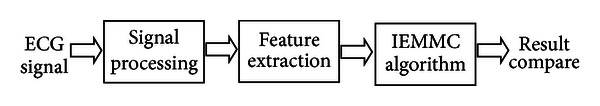
The automatic detection system for ECG arrhythmias.

The automatic detection system for ECG arrhythmias consists of three stages and is constructed as shown in Figure 1. The first stage is the preprocessing which includes filtering, baseline correction, and waveform detection. The second stage is the feature extraction which aims to find the best coefficients set to describe the ECG signal. The last stage is designed to cluster ECG periods using the IEMMC algorithm according to the previously extracted features in order to construct the arrhythmia classes.

### 2.1. Preprocessing

#### 2.1.1. ECG Signal Filtering

**Figure 2:**
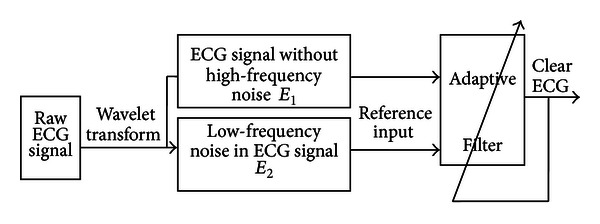
The adaptive ECG filter based on wavelet transforms.

ECG signals can be contaminated with several types of noise, such as motion artifact (MA), electromyogram noise (EMG), and baseline wandering (BW), which can affect the feature extraction algorithm. So, the ECG samples should be preprocessed before feature extraction and clustering. Due to the frequency spectrum overlapping between ECG signal and noise like motion artifact and baseline wandering which is less than 7 Hz, traditional wavelet decomposition and wavelet threshold method would make ECG waveform distorted, such as the distortion of *P* wave or *T* wave signal. For this situation, we apply a wavelet transform based adaptive filter which combines the advantages of wavelet transform and adaptive filtering techniques to preprocess the ECG signal. The construction of our ECG signal filter is demonstrated in Figure 2.

As Figure 2 shows, the procedures of the ECG signal filter can be summarized as the following four steps.

1. According to the sampling frequency of ECG signal, the least wavelet decomposition level *i* could be determined by separating ECG signal from high-frequency noise. Then, the ECG signal with noise could be wavelet decomposed into *i* scales.
2. After wavelet decomposition and removal of precise components containing high-frequency noise signal, we set the approximate components *E*_1_ which contain ECG signal without high-frequency noise as the primary input signal of the adaptive filter.
3. In line with spectrum relations between various waveform and low-frequency noise, such as baseline drift and motion artifact, the least wavelet decomposition level *j* which can separate ECG signal from low-frequency noise would be determined. By wavelet decomposition of *E*_1_ into *j* scales, the left approximate components *E*_2_ containing baseline drift, motion artifact, and other low-frequency interference would be taken as the reference input signal of the adaptive filter.
4. Least mean squares (LMS) adaptive filtering is used to preprocess the primary input signal and get clear ECG signals.

#### 2.1.2. Waveform Detection

The waveform detection of the ECG signal is the very basis of feature extraction. There are actually three separate algorithms, each of which is designated to detect certain waveform of ECG signal.

(1)  *R* *Detection*. The detection of *QRS* complex takes a vital role in ECG waveform detection. In order to achieve QRS complex detection, *R* wave must be located at first. According to the fact that *R* wave boasts the largest slope, difference of ECG amplitude array is generated to make *R* peaks more noticeable. Then, a practically lower limit is employed to remove unrelated noisy peaks from the signal. In order to avoid interference of big *T* wave, the relative refractory period, which lasts 200 ms after *R* peak is detected, should be skipped. Meanwhile, every *RR* interval should be judged in case of escaped inspection of *R* peak.

(2)  *QS Detection*. After finishing the positioning of *R* wave, *Q* and *S* peaks can be identified in accordance with the morphological characteristics. *Q* and *S* peaks occur around the *R* peak within 0.1 second. The turning point connecting baseline and falling edge is just the *Q* peak. Similarly, *S* peak could be found in the right side.

(3)  *P and T Wave Detection*. In the light of waveform characteristics of the normal ECG signal, it is found that *P* wave, *QRS* wave, and *T* wave appear alternately. Besides, the gap between the peak of *P* wave and *QRS* is no more than 0.16 seconds. This suggests that the maximum voltage point within 0.16 seconds before the *Q* peak shall be *P* peak, while the maximum voltage point between *S* peak and the next *P* peak shall be the *T* peak.

### 2.2. Feature Extraction

Feature extraction is a process to determine the best coefficients which could describe the ECG waveform accurately. In order to extract the best features that represent the structure of the ECG signals, nine times domain coefficients belonging to two succeeding ECG periods are considered, as shown in Table 1. The first row in the table is the name of the features, while the rest show the value of each feature. All features are listed as follows:

- normalized *RR* interval between the acquired *R* wave and the preceding *R* wave (*RR*_*n*_);
- normalized *RR* interval between the acquired *R* wave and the following *R* wave (*RR*_*n*_′);
- normalized *QRS* interval of the acquired beat (*QRS*_*n*_);
- normalized *PR* interval of the acquired beat (*PR*_*n*_);
- normalized *QT* interval belonging to the acquired beat (*QT*_*n*_);
- normalized *ST* interval of the acquired beat (*ST*_*n*_);
- normalized *R* amplitude of the acquired beat (*R*_*n*_);
- normalized *P* amplitude of the acquired beat (*P*_*n*_);
- normalized *T* amplitude of the acquired beat (*T*_*n*_).

*QR* *S* interval is calculated as the time interval between *Q* wave and *S* wave. *PR* interval is calculated as the time interval between the *P* peak and the *R* peak. *ST* interval is calculated as the time interval between *S* wave and *T* peak. *QT* interval is measured as the time interval between *T* wave and the onset time of the *Q* wave. From the medical point of view, the detection of arrhythmia depends on two or more ECG signal periods. The previous period of an ECG signal has many indicators of current arrhythmia. So, in our approach, two *QRS* periods' parameters *RR*_*n*_ and *RR*_*n*_′ are considered to be the features of ECG signal. *R* amplitude is measured as the distance between the peak of the *R* wave and the baseline. *P* amplitude and *T* amplitude are measured in the same way.

### 2.3. Clustering Method for ECG Arrhythmia

#### 2.3.1. Maximum Margin Clustering

The MMC extends the theory of SVM to the unsupervised scenario, which aims to find a way to label the samples by running SVM implicitly with the maximum margin over all possible labels [18].

Mathematically, given a point set *χ* = {*x*_1_,…, *x*_*n*_} and their labels *y* = {*y*_1_,…, *y*_*n*_} ∈ {−1,+1}^*n*^, SVM seeks a hyperplane *f*(*x*) = *w* ^*T*^ *ϕ*(*x*) + *b* by solving the following optimization problem:

(1) min⁡w,b,ξi⁡12||w||2+C∑i=1nξis.t. yi(wTϕ(x)+b)≥1−ξi,     ξi≥0,  i=1,…,n,

where *ϕ*(·) is a nonlinear function that maps the data samples in a high dimensional feature space and makes the nonseparable problem in the original data space to be separable in the feature space. The *ξ*_*i*_ values are called slack variables, and *C* > 0 is a manually chosen constant.

Different from SVM, where the class labels are given and the only variables are the hyperplane parameters (*w*, *b*), MMC aims at finding not only the optimal hyperplane (w*, b*), but also the optimal labeling vector *y* [17]. Originally, this task was formulated in terms of the following optimization problem [18]:

(2) min⁡y∈{−1,+1}n⁡min⁡w,b,ξi⁡12||w||2+C∑i=1nξis.t. yi(wTϕ(x)+b)≥1−ξi,  ξi≥0,  i=1,…,n,  C≥0.

However, the previous optimization problem has a trivially “optimal” solution, which is to assign all data to the same class and obtain an unbounded margin. Moreover, another unwanted solution is to separate a single outlier or a very small group of samples from the rest of the data. To alleviate these trivial solutions, Xu et al. [18] imposed a class balance constraint on *y*,

(3) $$\begin{array}{c} -\ell \leq e^{T}y\leq \ell , \end{array}$$

where *ℓ* ≥ 0 is a constant to control the class imbalance, which could bound the difference in class size and avoid assigning all patterns to the same class, and *e* is an all-one vector.

The MMC method often outperforms common clustering methods with respect to the accuracy [17, 18]. It can be expected that the detection of ECG arrhythmia by using the MMC algorithm will achieve a high level of accuracy. However, applying the approach requires solving a nonconvex integer problem, which is computationally expensive, and only small data sets can be handled by the MMC method so far. At present, various optimization techniques have been applied to handle this problem. Xu et al. [18] proposed to make several relaxations to the original MMC problem and reformulate it as a SDP problem, which can then be solved by standard SDP solvers such as SDPT3 and SeDuMi. Valizadegan and Jin [19] further proposed the generalized MMC algorithm which reduces the scale of the original SDP problem significantly. To make MMC method more practical, Zhang et al. [17] put forward a method which iteratively applied an SVM to improve an initial candidate obtained by a *K*-means preprocessing step. Recently, Zhao et al. [20] proposed a cutting plane MMC method based on constructing a sequence of intermediate tasks and each of the intermediate tasks, was solved using constrained concave-convex procedure. Although the recently proposed approaches have improved the efficiency of the MMC method, the application of these methods has not always been guaranteed. For example, as an iterative approach, the performance of iterSVR algorithm [17] which begins with assigning a set of initial labels is crucial for the quality of initialization. Random initialization will usually result in poor clustering.

#### 2.3.2. Maximum Margin Clustering with Immune Evolution

The concept of SVMs can be considered to be a special case of regularization problems in the following form:

(4) $$\begin{array}{c} \underset{f\in H}{\inf}\frac{1}{n}\sum_{i=1}^{n}L\left(y_{i},f\left(x_{i}\right)\right)+\lambda {||f||}_{H}^{2}, \end{array}$$

where *λ* > 0 is a fixed real number, *L* : *Y* × *ℜ* → [0, *∞*) is a loss function measuring the performance of the prediction function *f* on the training set, and ||*f*||_*H*_^2^ is the squared norm in a reproducing kernel Hilbert space *H*⊆*ℜ* ^*x*^ = {*f* : *Χ* → *ℜ*} induced by a kernel function. In the SVM approach (1), the hinge loss *L*_*h*_(*y*, *f*) = max⁡{0,1 − *yf*(*x*)} with *y* ∈ {−1, +1} is used. Instead of using the hinge loss, our approach penalizes overconfident predictions by using the square loss *L*_*s*_(*y*, *f*) = (*y* − *f*(*x*))^2^ leading to

(5) min⁡w,b,η12||w||2+C2∑i=1nη2s.t. yi((wTϕ(xi))+b)=1−η, i=1,…,n.

So, in our MMC algorithm, we aim at finding a solution for

(6) min⁡y∈{−1,+1}n,w,bJ(y,w,b)=12||w||2+C2∑i=1nη2s.t. yi((wTϕ(xi))+b)=1−η,      i=1,…,n,  −l≤∑i=1nyi≤l.

In order to solve problem (6), the original non-convex problem is considered to be a special case of optimization problem, and immune evolutionary algorithm is proposed to find optimal solution. Recent studies have shown that the immune evolutionary algorithm possesses several attractive immune properties that allow evolutionary algorithms to avoid premature convergence and improve local search capability [21–25]. By utilizing powerful global search capability and fast convergence of the immune evolutionary algorithm, IEMMC could avoid SDP relaxations and find optimal solution of the MMC method efficiently.

*The Process of IEMMC Algorithm*. The framework of our IEMMC algorithm is given by Algorithm 1.

**Algorithm 1 (Maximum Margin Clustering with Immune Evolution)**

* *

*Step *1. Generate a set of candidate solutions *P* = {*y*_1_,…, *y*_*m*+*r*_}⊆{−1,+1}^*n*^, composed of the subset of memory cells *P*_*m*_ added to the remaining *P*_*r*_(*P* = *P*_*m*_ + *P*_*r*_). *P* should fulfill the balance constraint (3) and ||*y*_*i*_ − *y*_*j*_|| > *t*_*s*_, *t*_*s*_ is the suppression threshold.

*Step *2. Compute the affinity values *F*(*y*) for each *y*_*j*_ ∈ *P*.

*Step *3. Determine the *N*_*c*_ best individuals, *P*_*c*_ of the population *P*_*r*_, based on an affinity measure. Perform clone selection on the population *P*_*c*_ to generate a temporary population of clones *P*_c_*.

*Step *4. Determine the *N*_*m*_ best individuals, *P*_*m*_ of the remaining population *P*_*r*_ − *P*_*c*_, based on an affinity measure. Apply mutation to the antibodies population *P*_*m*_, where the hypermutation is proportional to affinity of the antibody. A maturated antibody population *P*_m_* is generated.

*Step *5. Re-select the improved individuals from *P*_c_* and *P*_m_* to compose the memory set and the population *P*_*r*_.

*Step *6. Perform receptor editing, replace some low affinity antibodies of the population *P*_*r*_ by randomly created new antibodies, maintaining its diversity.

*Step *7. If termination conditions are not satisfied, go to Step 2.

*Step *8. Return the best individual *y*_*i*_.

The starting point is generating a set of candidate solutions *P* = {*y*_1_,…, *y*_*m*+*r*_}⊆{−1,+1}^*n*^, composed of the subset of memory cells *P*_*m*_ added to the remaining *P*_*r*_  (*P* = *P*_*m*_ + *P*_*r*_). Each of these individuals constitutes a possible solution for optimization problem (6). Throughout our IEMMC algorithm, we ensure that only valid individuals are created; that is, individuals *y* should fulfill the balance constraint (3). In Step 2, the affinity value *F*(*y*) is computed for each of the initial individuals, where

(7) $$\begin{array}{c} F\left(y\right)=\exp\left(-\min J\left(y,w,b\right)\right). \end{array}$$

Depending on the affinity values, the copies of the antibodies are generated, and clone selection is performed on superior individuals. In Step 4, mutation process is applied to the antibodies. If the affinity value of the new antibody is better than that of original value, new antibody is stored in the place of the original one; otherwise, old antibody is kept in population. After the mutation process, receptor editing is applied to the antibody population. In the receptor editing process, a percentage of antibodies in the antibody population are replaced by randomly created new antibodies. When the best individual satisfies termination condition, *y*_*i*_ would be returned.

*Fitness Computation*. For fixed solution *y*, the problem formulated in the function (6) could be solved by the standard SVM learning algorithm. So, we can compute (*w*, *b*) from the Karush-Kuhn-Tucker (KKT) conditions as usual to maximize margin between clusters. But this solution (*w*, *b*, *y*) is not the optimal clustering solutions for problem (6). Therefore, we continue to find a better bias *b* and cluster label *y* by fixing *w* and minimizing problem (6) which is reduced to

(8) min⁡y,b∑i=1n(w·ϕ(xi)+b−yi)2s.t. yi∈{±1}, i=1,…,n,  −ℓ≤eTy≤ℓ.

Then, problem (8) can be solved without the use of any optimization solver by the following proposition. At first, we sort *w* ^*T*^ *ϕ*(*x*_*i*_) and use the set of midpoints between any two consecutive w^*T*^ *ϕ*(*x*_*i*_) values as the candidates of *b*. From these candidates of *b*, the first (*n* − *l*)/2 and the last (*n* − *l*)/2 of the candidates should be removed for not satisfying the class balance constraint (3). For each remaining candidate, we determine *y* = sign⁡(*w* ^*T*^ *φ*(*x*) + *b*) and compute the corresponding objective value in (8). Finally, we choose *b* and corresponding *y* that has the optimal objective. Since both *w* and *b* have been determined, fitness value *F*(*y*) for the new individual *y* can be obtained by *F*(*y*) = exp⁡(−min⁡*J*(*y*, *w*, *b*)). 

## 3. Experiment and Results

### 3.1. Experimental Data

Experimental data of ECG arrhythmias used in this study are taken from MIT-BIH ECG Arrhythmias Database [26]. All ECG data are classified into five classes according to standard of The Association for the Advancement of Medical Instrumentation (AAMI) [27], since this database urges all users to follow the AAMI recommendations. In this standard, abnormal ECG could be divided into following four types. Type S contains atrial premature (AP), nodal premature (NP), and supraventricular premature (SP). Type V contains premature ventricular contraction (PVC) and ventricular ectopic (VE). Type F contains fusion of ventricular and normal beat. Type Q contains paced beat, fusion of paced and normal beat, and unclassified beat. The other kinds of heartbeats are considered as N type, including normal beat, atrial escape (AE), nodal escape (NE), right bundle branch block (R), and left bundle branch block (L).

Totally 1682 ECG periods are selected from seven records of MIT/BIH database to test the correctness of the IEMMC algorithm. The distribution of records is shown in Table 2. The first row corresponds to the labels according to the AAMI standard. And the first column is the name of the records, whereas the others contain the number of heartbeats of each type.

### 3.2. Experimental Results

In this section, we demonstrate the superiority of the proposed IEMMC procedure for ECG arrhythmias detection, and the following three types of performance evaluation indicators are used to assess the effect of ECG arrhythmias clustering method:

(9) $$\begin{array}{c} \text{sensitivity}=\frac{\text{TP}}{\left(\text{TP}+\text{FN}\right)},\\ \text{specificity}=\frac{\text{TN}}{\left(\text{FP}+\text{TN}\right)},\\ \text{accuracy}=\frac{\left(\text{TP}+\text{TN}\right)}{\left(\text{TP}+\text{FN}+\text{FP}+\text{TN}\right)}, \end{array}$$

where true positive (TP) means the number of true arrhythmia that has been successfully detected; false positive (FP) is the number of true arrhythmia that has been missed; true negative (TN) means the number of corresponding nontarget arrhythmia that has been correctly detected; false negative (FN) is the count of nontarget arrhythmia that has been detected wrongly. 

The simulation results are listed in Table 3, and the performance analysis of the clustering result is in Table 4. As shown in Tables 3 and 4, by using the IEMMC algorithm, the correctness of ECG arrhythmias is at a high level.

From the result, we can find that type N is the most regular and numerous heartbeats and easy to be separated from the other types; so, its result is better than other types. However, the performance of type F is lower than that in the previous case. Given that morphology of type F is often very similar to that of other types, it is very difficult to characterize type F.

**Figure 3:**
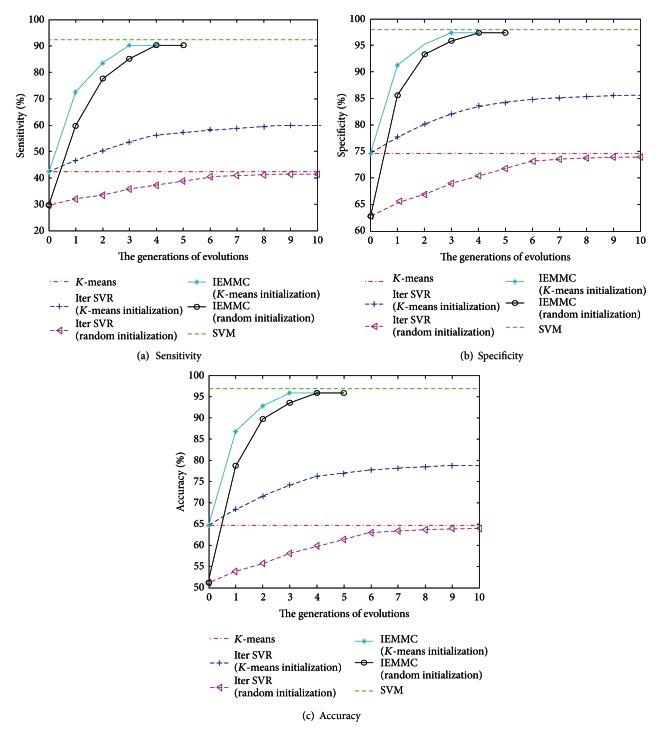
The performance comparison of different clustering methods.

In order to verify and measure the IEMMC algorithm's superiority, three methods are developed in parallel to compare with our algorithm, including standard *K*-means algorithm, iterSVR which is the first approach capable of dealing with large data sets [17], and SVM which has been proved to be a successful supervised learning method for ECG recognition and classification [8–11]. The performance of all clustering methods is shown in Figure 3. Two initialization schemes are developed for both iterSVR and IEMMC in the experiment: (1) random; (2) standard *K*-means clustering (KM). In the first scheme, initial candidate solutions of IEMMC and iterSVR are generated randomly. In the second scheme, iterSVR is initialized by standard *K*-means clustering. Only one of IEMMC candidate solutions is initialized by standard *K*-means clustering, and the rest solutions are generated at random. The class balance parameter of both IEMMC and iterSVR is always set as *L* = 0.2∗*n*. Also, 20% of the ECG data are extracted randomly to be the training data of the SVM classification. The radical basis function (RBF) kernel *k*(*x*, *x*′) = exp⁡(−||*x* − *x*′||/*σ* ^2^) is used for all the kernel methods in the experiment. As for the regularization parameter *C*, we choose the best value from a set of candidates (1, 10, 100, 500) for each data set. All algorithms are, respectively, repeated three times because of the inherent randomness. For each method and each data set, we report the result with its best value chosen from a set of candidates.

From Figure 3, the IEMMC's performance is as similar as that of the SVM and better than those of all clustering methods. Also, we can find that the performance of iterSVR largely depends on the superiority of initialization. With random initialization, clustering result from iterSVR is even worse than that of *K*-means algorithm. Since the performance of *K*-means is also unsatisfactory, even initialized by *K*-means, iterSVR still cannot meet the expectation of the ECG arrhythmia diagnosis. However, inheriting the outstanding global optimization ability of immune evolutionary algorithm, the IEMMC algorithm can find the best clustering for objective function in a very short evolution period, even in the case of random initialization. Additionally, IEMMC algorithm not only excelled in performance but also in convergence. While iterSVR needs to iterate ten times to find solution, the IEMMC algorithm only needs to evolve four generations. Especially, the IEMMC algorithm could obtain the same optimal solution from different initializations in few generations of evolutions, due to the prominent convergence and global search ability. This excellent performance in the experiment has proved that the IEMMC algorithm is very effective for the detection of ECG arrhythmia.

## 4. Conclusions

In this paper, a novel IEMMC algorithm is proposed to cluster the ECG signal and detect ECG arrhythmias, which iteratively updates the quality of candidates by means of immune evolutionary without employing any training process. The experimental analysis reveals that our approach yields better clustering performance than some competitive methods in most cases.

In the future, we will use some other biological principles based evolutionary algorithm to solve the MMC problem, like ant colony optimization and particle swarm optimizer, since they have been proved to have global optimizaton ability. Furthermore, comparison with immune evolutionary algorithm will be done to find out a more efficient ECG data clustering algorithm.

## Figures and Tables

**Table 1:** Nine features of ECG signal.

*RR*_*n*_ (s)	*RR*_*n*_′ (s)	*QR* *S*_*n*_ (s)	*PR*_*n*_ (s)	*QT*_*n*_ (s)	*ST*_*n*_ (s)	*R*_*n*_ (mv)	*P*_*n*_ (mv)	*T*_*n*_ (mv)
0.8477	0.8692	0.0742	0.1663	0.2930	0.2188	1.8149	0.0570	0.6817
0.9023	0.8931	0.0742	0.1445	0.2891	0.2148	1.6339	0.0142	0.5926
0.8594	0.8916	0.0781	0.1406	0.2852	0.2070	2.3085	0.0579	0.6125
0.8281	0.8034	0.0742	0.1663	0.2931	0.2109	2.1007	0.0469	0.6247

**Table 2:** The number of sample records according to arrhythmia type.

MIT code	N	S	V	F	Q	Total
106	104	0	83	0	0	187
200	125	0	112	0	0	237
208	95	0	0	86	0	181
209	102	106	0	0	0	208
213	106	0	0	113	0	219
217	205	0	0	0	211	416
222	122	112	0		0	234

Total	859	218	195	199	211	1682

**Table 3:** The ECG arrhythmias clustering results using the IEMMC algorithm.

	Clustering result				
Arrhythmia type	N	S	F	V	Q
N	803	15	12	13	16
S	27	191	0	0	0
V	35	0	164	0	0
F	17	0	0	178	0
Q	28	0	0	0	183

**Table 4:** The performance analysis result of the ECG arrhythmias clustering method.

Arrhythmia type	Sensitivity (%)	Specificity (%)	Accuracy (%)
N	97.9	92.7	95.4
S	83.0	98.0	95.8
F	82.4	97.5	95.6
V	82.8	98.7	96.6
Q	83.9	97.9	96.0

Total	90.3	97.4	95.9
